# The haemodynamic dilemma in emergency care: Is fluid responsiveness the answer? A systematic review

**DOI:** 10.1186/s13049-017-0370-4

**Published:** 2017-03-06

**Authors:** Mohammed H. Elwan, Ashraf Roshdy, Eman M. Elsharkawy, Salah M. Eltahan, Timothy J. Coats

**Affiliations:** 1 Department of Emergency Medicine, Alexandria University, Alexandria, Egypt; 2 Emergency Medicine Academic Group, Department of Cardiovascular Sciences, University of Leicester, Level G Jarvis Building RMO, Infirmary Square, LE1 5WW Leicester, UK; 3 Department of Critical Care Medicine, Alexandria University, Alexandria, Egypt; 4 General Intensive Care Unit, Broomfield hospital, Mid Essex NHS Trust, Chelmsford, UK; 5 Department of Cardiology, Alexandria University, Alexandria, Egypt

**Keywords:** Fluid therapy, Haemodynamics, Resuscitation, Cardiac output, Fluid responsiveness, Shock, Emergency

## Abstract

**Background:**

Fluid therapy is a common and crucial treatment in the emergency department (ED). While fluid responsiveness seems to be a promising method to titrate fluid therapy, the evidence for its value in ED is unclear. We aim to synthesise the existing literature investigating fluid responsiveness in ED.

**Methods:**

MEDLINE, Embase and the Cochrane library were searched for relevant peer-reviewed studies published from 1946 to present.

**Results:**

A total of 249 publications were retrieved of which 22 studies underwent full-text review and eight relevant studies were identified. Only 3 studies addressed clinical outcomes - including 2 randomised controlled trials and one feasibility study. Five articles evaluated the diagnostic accuracy of fluid responsiveness techniques in ED. Due to marked heterogeneity, it was not possible to combine results in a meta-analysis.

**Conclusion:**

High quality, adequately powered outcome studies are still lacking, so the place of fluid responsiveness in ED remains undefined. Future studies should have standardisation of patient groups, the target response and the underpinning theoretic concept of fluid responsiveness. The value of a fluid responsiveness based fluid resuscitation protocol needs to be established in a clinical trial.

## Background

Fluid therapy is a key part of resuscitation of the shocked patient in the Emergency Department (ED). Prompt and effective fluid resuscitation is required to prevent grave outcomes [1, 2]. However, excessive fluid has been associated with increased complications, length of hospital stay and mortality [3–7]. Titration of fluid therapy and the stratification of patients into those who will benefit and those who may be harmed is a clinical dilemma that confronts emergency physicians on a daily basis.

Fluid resuscitation is currently delivered with uncertainty and variability. There is uncertainty about the amount to give and when to stop. There is significant individual variation in the amount of fluid given between patients, especially in the elderly. This variation was evident in recent major clinical trials where the total volume of intravenous (IV) fluid therapy in ED varied from 200 ml to over 10,000 ml [8–10].

Pre-existing medical conditions or acute disease associated myocardial dysfunction alter the response to resuscitation. Critical care studies demonstrate that only half of haemodynamically unstable patients in intensive care units (ICUs) respond to fluid therapy [11]. It is likely that less than 40% of hypotensive patients with sepsis respond to fluid therapy [12, 13]. These patients can be harmed by excess fluid, as it may contribute to endothelial injury, organ dysfunction and increased morbidity and mortality [14–18]. ‘One-size-fits-all’ protocols are offered to initiate therapy [12], but do not solve the conundrums of continuing fluid management.

In ED, resuscitation remains largely guided by clinical examination, basic monitoring parameters (e.g. blood pressure and heart rate) and biochemical parameters (e.g. blood lactate), which are poor predictors of volume status [19, 20]. While change in central venous pressure (CVP) is still widely used, it has poor relationship with volume status [21, 22].

The immediate goal of fluid resuscitation is improving cardiac output (CO) and ultimately improving tissue perfusion. If a fluid bolus does not increase CO, it will not improve tissue perfusion and may be harmful. The haemodynamic response to fluid loading, ‘fluid responsiveness’ has been suggested as a dynamic guide for fluid therapy and a controlled method of resuscitation [23]. Testing fluid responsiveness involves both a fluid challenge, and subsequent monitoring of change in a haemodynamic parameter [18].

One suggested definition of fluid responsiveness is ‘an increase in a physiologic parameter, preferably cardiac output, within 15 min, superseding twice the error of the measuring technique after a 15-min administration of 6 mL/kg of crystalloids’ [24]. However there is little agreement on the type or amount of fluid to use, the rate of infusion or the physiological targets [25]. The main drawback of using a fluid challenge is that, if it is negative, fluid has been irreversibly administered to the patient.

Alternatively, the preload challenge can be by passive leg raise (PLR) [26] or positive pressure ventilation [27]. PLR has been used as a transient and reversible self-fluid challenge [26]. During PLR, recruitment of splanchnic and lower limb blood transfers a volume of around 300 mL of blood into the central circulation mimicking a fluid challenge [28]. The effect of PLR is transient reaching its maximum effect at approximately 1 min [29]. The final type of preload challenge is to use the circulatory effects of pressure cycles during mechanical ventilation, however this technique is not applicable to the majority of spontaneously breathing patients in ED [27].

A number of less invasive and noninvasive techniques for haemodynamic monitoring have been developed, which may be appropriate in emergency care. Less invasive methods include trans-oesophageal Doppler, transpulmonary thermodilution, pulse contour and pulse power analysis. Noninvasive methods include end-tidal carbon dioxide, transthoracic Doppler, bioimpedance, plethysmography and bioreactance [30].

Most of the evidence about different fluid responsiveness strategies comes from perioperative and critical care setting, however as anaesthesia and ventilation profoundly affect cardiovascular responses, these data are difficult to apply to the ED. There are no previous systematic reviews on this topic in ED. In this study, we aim to synthesise the existing literature investigating fluid responsiveness in in-hospital emergency care.

## Methods

A systematic review of literature was performed according to the Preferred Reporting Items for Systematic Reviews and Meta-Analyses (PRISMA) statement [31].

### PICO statement

Patient, In adult patients who may need fluid resuscitation in ED,

Intervention, Does testing fluid responsiveness

Comparison, Compared to standard care

Outcome, Affect haemodynamic or biochemical parameters, resource utilisation or mortality?

### Identification of records

We searched the US National Library of Medicine’s MEDLINE database via Ovid interface, Embase and the Cochrane library for relevant peer-reviewed studies published from 1946 to present. A clinical librarian assisted in the literature search.

**Fig. 1:**
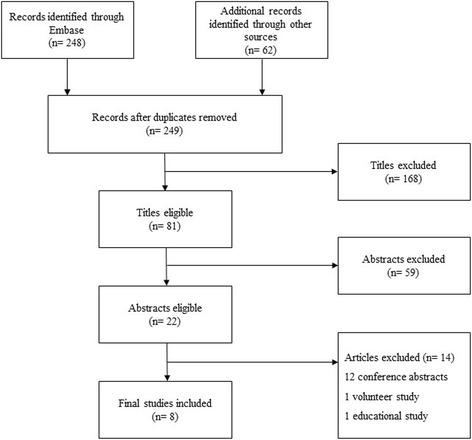
Screening process

We used the following search terms and subject headings: “emergency”, “fluid/preload or volume responsiveness” and “preload dependency” with appropriate variations where applicable (Table 1). The references of identified articles were used to identify further publications. An electronic search alert was set up to identify recently published studies. Retrieved results were transferred to Endnote® (Thomson Reuters) where duplicates were removed. Titles were screened for relevance initially, and abstracts were then further assessed. The full-text of eligible articles was obtained from NHS Athens and they were assessed for relevance. A flow diagram summarises the screening process (Fig. 1).

**Table 1:** Search strategy, Embase: 1974 – 20 June 2016

	Search term	Results
1	exp EMERGENCY HEALTH SERVICE/	77907
2	exp EMERGENCY MEDICINE/	33070
3	exp EMERGENCY WARD/	85683
4	“emergency department*”.ti,ab	87076
5	“emergency room*”.ti,ab	22819
6	(accident* adj2 emergenc*).ti,ab	5254
7	(“cardiac output*” adj2 increas*).ti,ab	6849
8	(“stroke volume*” adj2 increas*).ti,ab	2433
9	“emergency care”.ti,ab	7924
10	1 OR 2 OR 3 OR 4 OR 5 OR 6 OR 9	214830
11	“pre load dependen*”.ti,ab	7
12	“preload dependen*”.ti,ab	241
13	“fluid challeng*”.ti,ab	783
14	“fluid respon*”.ti,ab	1225
15	“pre load respon*”.ti,ab	0
16	“preload respon*”.ti,ab	117
17	“volume respon*”.ti,ab	1186
18	7 OR 8 OR 11 OR 12 OR 13 OR 14 OR 15 OR 16 OR 17	11672
19	10 AND 18	248

### Critical appraisal

Critical appraisal of the studies identified as relevant to the PICO question followed the CONSORT statement for randomized studies and the STARD checklist for diagnostic accuracy studies.[32, 33] Two authors (M.H.E and T.J.C) independently evaluated the methodologic quality of included studies and discussed the results. A third reviewer was available if a common rating was not agreed. Each study was rated according to the Oxford Clinical Evidence Based Medicine (OCEBM) levels of evidence [34].

## Results

A total of 249 publications were retrieved of which 22 studies underwent full-text review. Eight relevant studies were identified, with characteristics shown in Table 2, all of which were appraised as containing low level evidence. Twelve relevant conference abstracts were retrieved, however, we decided to exclude them as there was insufficient data to evaluate quality, and no subsequent full-text publications. Only 3 studies addressed clinical outcomes - including 2 randomised controlled trials (RCT) and one feasibility study (Table 3) [35–37]. Five articles evaluated the diagnostic accuracy of fluid responsiveness techniques in ED (Table 4) [38–42]. Neither diagnostic studies nor the feasibility study included a comparison to standard care. The two RCTs varied in patient groups, interventions and primary outcomes. Due to marked heterogeneity, it was not possible to combine results in a meta-analysis. In the following paragraphs, we elaborate on the findings of the two RCTs and the two highest quality diagnostic studies.

**Table 2:** General characteristics of included studies

Author	Year	Location	Setting	Design	Aims	Level of evidence*	Appraisal comments
Corl^38^	2012	USA	Single centre, academic ED	Diagnostic	To determine the accuracy of the caval index to detect fluid responsiveness	4	• High risk of bias • Convenience sample, unclear description of population • Gold standard (TEB) questionable • Patients were excluded from analysis due to incomplete data
Jung^42^	2012	Korea	Single centre, academic ED	Diagnostic	To determine the validity of corrected flow to predict fluid responsiveness	4	• High risk of bias • Not presented as a diagnostic test study • Unclear patient selection method • No blinding to reference test measurements. • No pre-specified threshold
Feissel^40^	2013	France	Single centre, ED	Diagnostic	To determine whether plethysmographic variability index can predict fluid responsiveness	3	• High risk of bias • Not presented as a diagnostic test study • Unclear patient selection method. • Inappropriate exclusions • Patients were excluded from analysis due to incomplete data • No pre-specified threshold
Coen^37^	2014	Italy	Single centre, major metropolitan ED	Treatment	To investigate the reliability of caval index and lung ultrasound to guide fluid infusion	4	• High risk of bias • No control group • No sample size calculation
de Valk^39^	2014	Netherlands	Single centre, academic ED	Diagnostic	To investigate the reliability of caval index to predict fluid responsiveness	4	• High risk of bias • Convenience sample • No pre-specified threshold. • Gold standard test questionable • No blinding to index test measurements • Patients were excluded from analysis due to incomplete data • No pre-specified threshold
Duus^41^	2015	USA	Single centre, adult ED	Diagnostic	To determine the reproducibility of PLR and fluid bolus monitored by bioreactance in predicting fluid responsiveness	3	• High risk of bias • Observational • Convenience sample. • Consecutive measures may influence each other • No blinding to index test measurements
Hou^36^	2016	USA	Multi centre	Treatment (RCT)	To evaluate the impact of a fluid responsiveness protocol in decreasing organ failure	4	• Lack of blinding • Relatively few patients per center • Only collected 10% of planned sample. Very likely Type II error • Downgraded due to imprecision
Kuan^35^	2016	Singapore	Single centre, academic ED	Treatment (RCT)	To Evaluate a non-invasive heamodynamic algorithm compared to standard care	2	• Lack of blinding • Underpowered: high difference in primary outcome – ARR 25%. RRR approx. 40%

RCT, Randomised controlled trial; TEB, thoracic electrical bioimpedance; ARR, absolute risk reduction; RRR, relative risk reduction; ED, emergency department; PLR, passive leg raise

**Table 3:** Main characteristics of clinical studies

Author	Participants					Intervention					Control	Primary outcome
	Inclusion criteria	n	SOFA	Basline MAP	Baseline lactate	Dynamic test	Monitor	Parameter measured	Cutoff	Treatment		
Kuan^35^	Age ≥21, two SIRS criteria, suspected infection and lactate ≥ 3 mmol/L	122	3.4 ± 2.6*	88 ± 18*	4.8 ± 2*	PLR	Bioreactance	∆SVI	10%	500 mL bolus	Standard care (target MAP > =65)	Rate of lactate clearance >20% at 3 h
									20%	1 L bolus		
Hou^36^	Age ≥18, two SIRS criteria, lactate ≥2 mmol/L and < 4 mmol/L and 4 h from ED presentation	64	1.1 ± 1.5*	92*	2.6 ± 0.4*	Fluid bolus (5 ml/kg over 10 min)	Bioreactance	∆SV (within 5 min of the fluid bolus)	10%	1 L infusion	Standard care	Worsening SOFA score by > =1 point over the first 72 h
Coen^37^	Age ≥18, Septic shock or Lactate >4 mmol/L	51	7.1 ± 2.9	60 ± 13	4.6 ± 3.3	Spontaneous breathing	Ultrasound	Caval index	30%	500 mL bolus	N/A	N/A

*Combined from treatment and control groups estimates

SIRS, systemic inflammatory response syndrome; SOFA, sequential organ failure assessment; MAP, mean arterial pressure; PLR, passive leg raise; SVI, stroke volume index; SV, stroke volume

**Table 4:** Summary of diagnostic accuracy studies

Author	Participants						Intervention				Criterion standard (FR definition)	Rate of FR (%)	SN	SP	AUC
	Inclusion criteria	No.	Disease severity	Base BP	Base Lactate	Spont. breathing	Dynamic test	Monitor	Parameter measured	Cutoff					
Corl^38^	Age ≥18, clinically expected to be eu- or hypovolaemic	26	N/A	SBP 114	N/A	Yes	PLR	U/S	∆ Caval index	N/A	≥10% increase in CI by TEB 4 min after PLR	31	N/A	N/A	0.56 (0.31–0.81)
Duus^41^	Age ≥18 and decided to receive a fluid bolus	109 (100 were analysed)	APACHE II 5 ± 3.6	MAP 126 ± 19	1.4 ± 0.6	Yes	PLR	Bioreactance	∆SV	10%	≥10% increase in SV by bioreactance after a fluid bolus (at least 5 ml/kg – no pre-defined infusion time or time frame for assessment)	60	80% (72%-88%)	61% (51%-71%)	N/A
de Valk^39^	At least one clinical sign of shock and decided to receive a fluid bolus	52 (45 were analysed)	N/A	MAP 73.8 ± 14.2	1,9 ± 1.1	Yes	Spontaneous breathing	U/S	Caval Index	36.5% (Not pre-defined)	≥10 mmHg increase in BP 15 min after a fluid bolus (500 ml NaCl 0.9% over 15 min)	27	83%	67%	0.741
Feissel^40^	Age ≥18, mechanically ventilated, sinus rhythm and septic shock	39 (31 were analysed)	SOFA 14 ± 2	72 ± 19*	N/A	No	MV	Plethysmography	Plethysmographic variability index (PVI)	19% (Not pre-defined)	≥15% increase in VTI by TTE 5 min after a fluid bolus (8 mL/kg HES over 20 min)	52	94% (69%-100%)	87% (61%-97%)	0.97 (0.83-0.99)
Jung^42^	Spontaneous breathing, sepsis-induced hypotension	26 (22 were analysed)	N/A	N/A	N/A	Yes	None	Oesophageal Doppler	Baseline corrected flow time (FTc)	301 ms (Not pre-defined)	≥10% increase in SVI by oesophageal Doppler immediately after a fluid bolus (7 mL/kg HES over 30 min)	65	88.2%	88.8%	0.870 (0.708–0.979)

*Combined from responders and non-responders estimates

BP, blood pressure; FR, fluid responsiveness; SN, sensitivity; SP, specificity; AUC, area under the curve; PLR, passive leg raise; SVI, stroke volume index; SV, stroke volume; U/S, ultrasound; SOFA, sequential organ failure assessment; SBP, systolic blood pressure; MAP, mean arterial pressure; APACHE, Acute Physiology and Chronic Health Evaluation; MV, mechanical ventilation; CI, cardiac index; VTI, velocity time integral; TTE, trans-thoracic echocardiography; HES, hydroxylethyl starch

Kuan et al.[35] compared a fluid responsiveness protocol to standard care. Inclusion criteria were sepsis (defined by two systemic inflammatory response syndrome, SIRS, criteria and suspected infection) and lactate ≥3 mmol/L. Fluid responsiveness was estimated by change in stroke volume index (∆SVI) measured by bioreactance CO monitor after PLR. A positive response (defined as 10%–20% ∆SVI) triggered infusion of 500 mL or 1 L of crystalloids respectively. The primary outcome was lactate clearance of more than 20% at 3 h. Initial fluid responsiveness was observed in 79% of treatment group patients. The treatment group received clinically significant more IV fluid (975 mL; 95% CI−450 to 1,725 mL) by the end of study at 3 h, but there was no significant difference in the rate of lactate clearance at 3 h or in-hospital mortality. Both groups received comparable amount of fluid at 24 h. In a planned subgroup analysis of patients with pre-existing fluid overload (e.g. congestive heart failure and renal failure), fluid responsiveness group received more fluid at 3 h.

Hou et al. [36] compared standard care to a fluid responsiveness protocol using a fluid bolus and bioreactance CO monitor. Inclusion criteria were age ≥18, two SIRS criteria, lactate ≥2 mmol/L and < 4 mmol/L and 4 h from ED presentation. Fluid responsiveness, defined as >10% increase in stroke volume (SV) after a 5 mL/Kg fluid bolus, mandated 1 L of fluid infusion. The primary outcome was change of sequential organ failure assessment (SOFA) score ≥1 over 72 h. The rate of fluid responsiveness was 47%. There was no significant difference in increase in SOFA score, the rate of hospitalisation or change in lactate over 4 h. The treatment group received significantly more IV fluid at 4 h 2633 cc (2264 – 3001) vs 1002 cc (707 – 1298), however the amount of fluid received was similar at 24 h.

Duus et al. [41] evaluated the diagnostic accuracy of PLR compared to fluid bolus - both measured by bioreactance. Fluid responsiveness was defined as ≥10% increase in SV after a fluid bolus. The rate of fluid responsiveness was 60%. PLR predicted fluid responsiveness with a sensitivity of 80% (72%–88%) and specificity of 61% (51%–71%). All patients were spontaneously breathing and most were of low disease severity (Table 4).

Feissel et al. [40] evaluated Plethysmographic variability index (PVI) during mechanical ventilation compared to an 8 mL/kg hydroxylethyl starch bolus. Fluid responsiveness was defined as ≥15% increase in velocity time integral (VTI) by echocardiography after the fluid bolus. Fluid responsiveness was detected in 52% of patients. PVI predicted the response to fluid loading with a sensitivity of 94% (69%–100%), specificity of 87% (61%–97%) and area under the curve (AUC) of 0.97 (0.83–0.99) (Table 4).

### Limitations

This is the first review of fluid responsiveness in emergency medicine, and has a number of limitations. This review was not registered in advance, which may increase the risk of reporting bias. Literature search was undertaken by one author (M.H.E.), however, a clinical librarian was involved in the search strategy. We used wide search criteria from three sources and hand searched references to include relevant studies, however we excluded conference abstracts that could not be assessed for methodological quality.

## Discussion

While fluid responsiveness is directly relevant to emergency care, we have found very little evidence (489 patients in total) on which to base best practice. This small number of studies reflects a relatively new concept to emergency care compared to anaesthesia and critical care - where fluid therapy has evolved over many years [11, 43]. Most studies suffered from methodological limitations and there was a large amount of heterogeneity, which makes interpretation difficult.

The main sources of heterogeneity were (1) the patient groups, (2) the type of preload challenge, (3) the monitoring method used, and (4) the definition of ‘fluid responsive’.

### Patients

Patients included in the studies had a wide range of underlying conditions of varying severity. In contrast with ICU and peri-operative studies [44], most patients were spontaneously breathing (except the Feissel et al. [40] study of mechanically ventilated patients in ED). The 2 RCTs were based on less severe patients in early sepsis, with both studies having low generalizability as they excluded patients with a wide range of comorbid conditions. In both studies patient inclusion hinged mainly on the diagnosis of sepsis and elevated lactate. However, intensive fluid therapy based on lactate alone may not be appropriate [45]. There is no clear signal from the evidence about whether future research should concentrate on high risk or lower risk patients.

### Preload challenge

To give a preload challenge three studies used PLR [35, 38, 41], one study used a fluid challenge, [36] two used the spontaneous breathing effect [37, 40],one used mechanical ventilation [40] and one did not use a preload challenge and relied on baseline measurements [42]. These methods are not necessarily comparable, and there is little evidence about which is best in ED.

Fluid bolus has been used as the criterion method (gold standard) of preload challenge in all but one diagnostic studies [39–42]. The fluid challenge varied in type, amount, infusion time and time frame for assessment (Table 4). Similar variability was reported in ICU practice [46] and in similar diagnostic studies where fluid challenge was used as a criterion standard [44, 47]. A recent review showed heterogeneity in fluid bolus therapy and a lack of studies correlating the physiological effects of a fluid bolus to clinical outcomes [25].

### Monitoring

Most of the included studies tested non-invasive monitors to track CO (or one of its surrogates). This is in line with a recent French survey that demonstrated that ED clinicians prefer to use the less invasive and less time-consuming indicators of fluid responsiveness [48]. In our review bioreactance was employed in 3 studies [35, 36, 41], ultrasound derived caval index in 3 studies [37–39] and plethysmography in one study [40]. One study used oesophageal Doppler in the ED, but this is probably too invasive to be generalizable [42].

In the diagnostic accuracy studies (Table 4), the variation in definition of the criterion standard made comparison difficult. Two studies utilised the same monitor for both the test and reference methods [41, 42] (one using bioreactance and the other oesophageal Doppler) violating the assumption of independence and leading to risk of overestimation of effect [41, 42]. The remaining 3 studies each used a different reference test, (one each using bioimpedance, non-invasive blood pressure and trans-thoracic echocardiography).

### Fluid responsiveness definition

There was a variety of definitions used to define ‘fluid responsive’. The commonest definition of fluid responsiveness was a 10% increase in SV, cardiac index (CI) or SVI (5 studies) [35, 36, 38, 41, 42]. Caval index, blood pressure and echocardiographic velocity time integral (VTI) were used in the remaining 3 studies. Using these different definitions and the varying entry criteria the rate of fluid responsiveness in the patients studied ranged from 31% to 79%.

Previous studies, mostly in ICU, used >0 to 20% change in SV or CO as a threshold for fluid responsiveness [11]. More recently this range has narrowed with a 10–15% change commonly used to define a change that is not due to random variability [49]. This cut-off is sometimes derived from the reported precision of thermodilution [50], and may not be optimal when a different device is used [51]. The clinical significance of a given change should be also appreciated.

### Diagnostic accuracy

The diagnostic accuracy of different techniques varied greatly (Table 4). This may be explained by the heterogeneity of subjects and methods or by true differences between the techniques. Notably, the highest accuracy was observed in mechanically ventilated patients, [40] which may show the technical challenge of applying fluid responsiveness to spontaneously breathing and moving patients in ED.

The diagnostic accuracy of different fluid responsiveness predictors have been more thoroughly studied in ICU and peri-operative setting. PLR-induced changes in CO has been validated against a ‘gold standard’ fluid bolus in several meta-analyses [44, 47, 52, 53]. It showed a reasonable accuracy regardless of the monitoring method or ventilation mode (sensitivity 85–89%, specificity 91–92% and area under the curve 0.95). Lower accuracy was observed for caval index, especially in spontaneously breathing patients - with a higher cut-off value to define a positive response [54].

In ED patients, spontaneous breathing, arrhythmia and autonomic response to PLR may confound the validity of the test. The test may also be different in ED patients who may be more hypovolaemic in the early stages of treatment, compared to the more fluid replete patients in ICU. The accuracy of the caval index remains questionable in spontaneously breathing patients [54], and the methods based on mechanical ventilation (e.g. plethysmography) are only relevant to a few of the most severely ill ED patients [55].

### Outcomes

One of the three clinical trials was a feasibility study without a primary outcome. The remaining two studies used different primary outcomes – lactate clearance and SOFA score change (Table 3). Both studies reported no significant difference in primary outcomes. However Type II error is possible, as the trial that used a clinically important endpoint (SOFA score at 72 h) only recruited 10% of the planned sample size, so the results cannot be interpreted [36].

A secondary outcome for all studies was the amount of fluid given. The fluid responsiveness based protocols triggered more intensive early resuscitation, however the total fluid received by fluid responsiveness and standard care groups seemed to equalise by the end of the first 24 h. So, total fluid volume was the same, but the intervention meant that the fluid was ‘front-loaded’ into the early phase of care. A similar effect has been seen in the recent major protocolised sepsis care trials [8–10, 56].

There are limited data on the usefulness of the fluid responsiveness approach to fluid management. A 2012 Cochrane review showed no mortality benefit from flow-guided haemodynamic approach in the peri-operative setting. This was supported by a recent RCT of a CO-guided haemodynamic algorithm (repeated 250 ml fluid challenges over 5 min. Fluid responsiveness was defined as ≥10% increase in SV sustained for 20 min or more) [57]. There was no significant difference in morbidity or mortality. However, the authors went on to include their findings to an updated meta-analysis of 37 more trials and found fewer complications in the intervention arm.

### Implications and uncertainties

The clinical relevance of ‘fluid responsiveness’ in the ED may be similar in principle to other settings. However, fluid management in ED is faced with unique challenges (e.g. early disease, elderly patients), in addition to limited time, limited resources and sometimes delay in transfer to intensive care. Hence, studies in other settings may not resolve the ED haemodynamic dilemma. As the benefit of fluid administration is often related to early administration, we think that, despite the practical difficulties, studying fluid resuscitation in the ED is fundamental for improving patient outcomes. Fluid responsiveness based resuscitation strategies have not been adequately tested in the ED to know whether or not they influence outcome, but fluid responsiveness remains an attractive concept.

We were unable to perform any meta-analysis due to the large variation within the literature. To resolve the current heterogeneity, we would like to suggest some standardisation for future research:

- *Which patient group?*
- Standardised definition of the target patient group is required, and the ‘uncertainty principle’ could be used. It is unreasonable to delay fluid administration for overtly hypovolaemic or septic patients, and a fluid responsiveness test is not needed. It is also unreasonable to give fluid if the clinician thinks that this may be harmful. However, in patients where the clinician is uncertain about the best course of action a test of fluid responsiveness may help. Future research should target the groups where there is clinical uncertainty.
- *What is the target response?*
- It is difficult to interpret a diagnostic test when the target response is ill defined. Current theory suggests that fluid responsiveness is a normal state (normal hearts are functioning on the ascending limb of the Frank-Starling curve) [23, 58]. However, only about half of healthy volunteers show a positive fluid test response [59–61]. Other studies have shown that healthy supine adults do not increase their SV after a preload challenge [62, 63]. So either the test is not measuring what we think that it is measuring (other factors also influencing the test result) or the theoretic concept of ‘normality’ on the Frank-Starling curve is an over-simplification of complex cardiovascular physiology. It is therefore not correct (and a source of confusion in the current literature) to think of “responsiveness” as a target. Future studies should use an endpoint that is independent of the fluid responsiveness test.
- *What outcome should be used?*
- The outcome should be independent of the test and relevant to the patient. Survival (lived/died) is appropriate for large scale interventional clinical trials of a fluid responsiveness based resuscitation protocols. Surrogate outcomes should be carefully assessed for patient relevance. We would suggest that ‘volume of fluid used’ does not pass this test and should not be used as a primary outcome in future studies.
- *When to start and when to stop fluid resuscitation?*
- Fluid responsiveness does not tell us when to start or stop fluid therapy (about 50% of volunteers would have received unnecessary fluid based on a positive response). The human volunteer studies suggest that it may be unwise to use a fluid responsiveness test as a definition of the resuscitated state (an endpoint for resuscitation). A fluid responsiveness test may help the clinician decide if more fluid could help achieve the resuscitated state (by predicting the effect of a fluid bolus), but cannot be used to define when the resuscitated state has been achieved (for which there are already well established clinical parameters). Future studies should not use fluid responsiveness to determine when to start or stop fluid resuscitation.
- *What is the conceptual framework for fluid responsiveness in ED?*
- Much of the current heterogeneity within the literature seems to be based on different concepts of how a fluid responsiveness test could potentially be used in an ED management protocol. The key concept is that the test is only a predictor of the haemodynamic effect of further fluid resuscitation – the clinician still has to decide whether further resuscitation is required and incorporate the fluid response prediction into the wider picture of the patient’s physiological state. Future studies would have less variation if based around this central concept.

## Conclusion

One-size-fits-all protocolised ‘fixed goal’ care has been challenged [8–10] and there is more interest in individualised care. The use of fluid responsiveness was investigated in the ED in 8 articles with considerable methodological heterogeneity. There were no high quality, adequately powered outcome studies so the place of fluid responsiveness in ED remains undefined. Future studies should have standardisation of patient groups, the target response and the underpinning theoretic concept of fluid responsiveness. The value of a fluid responsiveness based fluid resuscitation protocol in the ED needs to be established in a clinical trial.

## References

[1] Angus DC, Van der Poll T (2013). Severe sepsis and septic shock. N Engl J Med.

[2] Leisman D, Wie B, Doerfler M, Bianculli A, Ward MF, Akerman M (2016). Association of fluid resuscitation initiation within 30 minutes of severe sepsis and septic shock recognition with reduced mortality and length of stay. Annals of emergency medicine.

[3] Shapiro NI, Howell MD, Talmor D, Lahey D, Ngo L, Buras J, Wolfe RE, Weiss JW, Lisbon A (2006). Implementation and outcomes of the Multiple Urgent Sepsis Therapies (MUST) protocol*. Crit Care Med.

[4] Jones AE, Brown MD, Trzeciak S, Shapiro NI, Garrett JS, Heffner AC, Kline JA (2008). The effect of a quantitative resuscitation strategy on mortality in patients with sepsis: a meta-analysis. Crit Care Med.

[5] Jones AE, Shapiro NI, Trzeciak S, Arnold RC, Claremont HA, Kline JA (2010). Investigators EMSRN: Lactate clearance vs central venous oxygen saturation as goals of early sepsis therapy: a randomized clinical trial. Jama.

[6] Boyd JH, Forbes J, Nakada T-a, Walley KR, Russell JA (2011). Fluid resuscitation in septic shock: A positive fluid balance and elevated central venous pressure are associated with increased mortality*. Crit Care Med.

[7] Maitland K, Kiguli S, Opoka RO, Engoru C, Olupot-Olupot P, Akech SO, Nyeko R, Mtove G, Reyburn H, Lang T (2011). Mortality after fluid bolus in African children with severe infection. N Engl J Med.

[8] Mouncey PR, Osborn TM, Power GS, Harrison DA, Sadique MZ, Grieve RD, Jahan R, Harvey SE, Bell D, Bion JF (2015). Trial of early, goal-directed resuscitation for septic shock. N Engl J Med.

[9] Yealy DM, Kellum JA, Huang DT, Barnato AE, Weissfeld LA, Pike F, Terndrup T, Wang HE, Hou PC, LoVecchio F (2014). A randomized trial of protocol-based care for early septic shock. N Engl J Med.

[10] Peake SL, Delaney A, Bailey M, Bellomo R, Cameron PA, Cooper DJ, Higgins AM, Holdgate A, Howe BD, Webb S (2014). Goal-directed resuscitation for patients with early septic shock. N Engl J Med.

[11] Michard F, Teboul J-L (2002). Predicting fluid responsiveness in ICU patients: a critical analysis of the evidence. CHEST Journal.

[12] Dellinger RP, Levy MM, Rhodes A, Annane D, Gerlach H, Opal SM, Sevransky JE, Sprung CL, Douglas IS, Jaeschke R (2013). Surviving Sepsis Campaign: international guidelines for management of severe sepsis and septic shock, 2012. Intensive Care Med.

[13] Lammi MR, Aiello B, Burg GT, Rehman T, Douglas IS, Wheeler AP, deBoisblanc BP (2015). Response to fluid boluses in the fluid and catheter treatment trial. Chest.

[14] Wang C-H, Hsieh W-H, Chou H-C, Huang Y-S, Shen J-H, Yeo YH, Chang H-E, Chen S-C, Lee C-C (2014). Liberal versus restricted fluid resuscitation strategies in trauma patients: a systematic review and meta-analysis of randomized controlled trials and observational studies*. Crit Care Med.

[15] Micek ST, McEvoy C, McKenzie M, Hampton N, Doherty JA, Kollef MH (2013). Fluid balance and cardiac function in septic shock as predictors of hospital mortality. Crit Care.

[16] Jozwiak M, Silva S, Persichini R, Anguel N, Osman D, Richard C, Teboul J-L, Monnet X (2013). Extravascular lung water is an independent prognostic factor in patients with acute respiratory distress syndrome*. Crit Care Med.

[17] Payen D, de Pont AC, Sakr Y, Spies C, Reinhart K, Vincent JL (2008). A positive fluid balance is associated with a worse outcome in patients with acute renal failure. Crit Care.

[18] Marik PE (2014). Early management of severe sepsis: concepts and controversies. Chest.

[19] Maurer C, Wagner JY, Schmid RM, Saugel B. Assessment of volume status and fluid responsiveness in the emergency department: A systematic approach. Medizinische Klinik-Intensivmedizin und Notfallmedizin. 2015;16:1–8. 10.1007/s00063-015-0124-x 26676240

[20] Saugel B, Ringmaier S, Holzapfel K, Schuster T, Phillip V, Schmid RM, Huber W (2011). Physical examination, central venous pressure, and chest radiography for the prediction of transpulmonary thermodilution-derived hemodynamic parameters in critically ill patients: a prospective trial. J Crit Care.

[21] Marik PE, Cavallazzi R (2013). Does the central venous pressure predict fluid responsiveness? An updated meta-analysis and a plea for some common sense. Crit Care Med.

[22] Marik PE, Baram M, Vahid B (2008). Does central venous pressure predict fluid responsiveness?*: A systematic review of the literature and the tale of seven mares. Chest.

[23] Marik PE, Lemson J (2014). Fluid responsiveness: an evolution of our understanding. Br J Anaesth.

[24] Cherpanath TG, Aarts LP, Groeneveld JA, Geerts BF (2014). Defining fluid responsiveness: a guide to patient-tailored volume titration. J Cardiothorac Vasc Anesth.

[25] Glassford NJ, Eastwood GM, Bellomo R (2014). Physiological changes after fluid bolus therapy in sepsis: a systematic review of contemporary data. Crit Care.

[26] Monnet X, Teboul J-L (2015). Passive leg raising: five rules, not a drop of fluid!. Crit Care.

[27] Monnet X, Teboul J-L (2013). Assessment of volume responsiveness during mechanical ventilation: recent advances. Crit Care.

[28] Lafanechere A, Pene F, Goulenok C, Delahaye A, Mallet V, Choukroun G, Chiche JD, Mira JP, Cariou A (2006). Changes in aortic blood flow induced by passive leg raising predict fluid responsiveness in critically ill patients. Crit Care.

[29] Monnet X, Rienzo M, Osman D, Anguel N, Richard C, Pinsky MR, Teboul JL (2006). Passive leg raising predicts fluid responsiveness in the critically ill. Crit Care Med.

[30] Marik PE (2013). Noninvasive cardiac output monitors: a state-of the-art review. J Cardiothorac Vasc Anesth.

[31] Moher D, Liberati A, Tetzlaff J, Altman DG (2009). Preferred reporting items for systematic reviews and meta-analyses: the PRISMA statement. Ann Intern Med.

[32] Schulz KF, Altman DG, Moher D (2010). CONSORT 2010 statement: updated guidelines for reporting parallel group randomised trials. BMC Med.

[33] Bossuyt PM, Reitsma JB, Bruns DE, Gatsonis CA, Glasziou PP, Irwig L, Lijmer JG, Moher D, Rennie D, De Vet HC (2015). STARD 2015: an updated list of essential items for reporting diagnostic accuracy studies. Radiology.

[34] OCEBM Levels of Evidence Working Group. “The Oxford 2011 Levels of Evidence”. Oxford Centre for Evidence-Based Medicine. http://www.cebm.net/index.aspx?o=5653. Accessed 8 Oct 2016.

[35] Kuan WS, Ibrahim I, Leong BS, Jain S, Lu Q, Cheung YB, Mahadevan M (2016). Emergency Department Management of Sepsis Patients: A Randomized, Goal-Oriented, Noninvasive Sepsis Trial. Ann Emerg Med.

[36] Hou PC, Filbin MR, Napoli A, Feldman J, Pang PS, Sankoff J, Lo BM, Dickey-White H, Birkhahn RH, Shapiro NI. Cardiac output monitoring managing intravenous therapy (COMMIT) to treat emergency department patients with sepsis. Shock (Augusta, Ga.). 2016;46(2):132. 10.1097/SHK.0000000000000564 PMC4957967 26925867

[37] Coen D, Cortellaro F, Pasini S, Tombini V, Vaccaro A, Montalbetti L, Cazzaniga M, Boghi D (2014). Towards a less invasive approach to the early goal-directed treatment of septic shock in the ED. Am J Emerg Med.

[38] Corl K, Napoli AM, Gardiner F (2012). Bedside sonographic measurement of the inferior vena cava caval index is a poor predictor of fluid responsiveness in emergency department patients. Emerg Med Australas.

[39] de Valk S, Olgers TJ, Holman M, Ismael F, Ligtenberg JJ, Ter Maaten JC (2014). The caval index: an adequate non-invasive ultrasound parameter to predict fluid responsiveness in the emergency department?. BMC Anesthesiol.

[40] Feissel M, Kalakhy R, Banwarth P, Badie J, Pavon A, Faller JP, Quenot JP (2013). Plethysmographic variation index predicts fluid responsiveness in ventilated patients in the early phase of septic shock in the emergency department: a pilot study. J Crit Care.

[41] Duus N, Shogilev DJ, Skibsted S, Zijlstra HW, Fish E, Oren-Grinberg A, Lior Y, Novack V, Talmor D, Kirkegaard H, Shapiro NI (2015). The reliability and validity of passive leg raise and fluid bolus to assess fluid responsiveness in spontaneously breathing emergency department patients. J Crit Care.

[42] Jung SM, Ryu S, Cho YC, Lee SH, Lim JS, Yun SY, Yoo IS (2012). Validity of corrected flow time (FTC) as a predictor of fluid responsiveness in patients with sepsis-induced hypotension. J Korean Soc Emerg Med.

[43] Ansari B, Zochios V, Falter F, Klein A (2016). Physiological controversies and methods used to determine fluid responsiveness: a qualitative systematic review. Anaesthesia.

[44] Cherpanath TG, Hirsch A, Geerts BF, Lagrand WK, Leeflang MM, Schultz MJ, Groeneveld AB (2016). Predicting Fluid Responsiveness by Passive Leg Raising: A Systematic Review and Meta-Analysis of 23 Clinical Trials. Crit Care Med.

[45] Bakker J, Nijsten MW, Jansen TC (2013). Clinical use of lactate monitoring in critically ill patients. Ann Intensive Care.

[46] Cecconi M, Hofer C, Teboul J-L, Pettila V, Wilkman E, Molnar Z, Della Rocca G, Aldecoa C, Artigas A, Jog S (2015). Fluid challenges in intensive care: the FENICE study. Intensive Care Med.

[47] Monnet X, Teboul J (2015). Passive leg raising for predicting fluid responsiveness: a systematic review and meta-analysis. Intensive Care Med Exp.

[48] Melot J, Sebbane M, Dingemans G, Claret PG, Arbouet E, Barkat B, Jamet P, Kovalevsky P, Louart B, Moreau A (2012). Use of indicators of fluid responsiveness in septic shock: a survey in public emergency departments. Ann Fr Anesth Reanim.

[49] Cecconi M, De Backer D, Antonelli M, Beale R, Bakker J, Hofer C, Jaeschke R, Mebazaa A, Pinsky MR, Teboul JL (2014). Consensus on circulatory shock and hemodynamic monitoring. Task force of the European Society of Intensive Care Medicine. Intensive Care Med.

[50] Stetz CW, Miller RG, Kelly GE, Raffin TA (1982). Reliability of the Thermodilution Method in the Determination of Cardiac Output in Clinical Practice 1, 2. Am Rev Respir Dis.

[51] Montenij LJ, Buhre WF, Jansen JR, Kruitwagen CL, de Waal EE (2016). Methodology of method comparison studies evaluating the validity of cardiac output monitors: a stepwise approach and checklist. Br J Anaesth.

[52] Cavallaro F, Sandroni C, Marano C, La Torre G, Mannocci A, De Waure C, Bello G, Maviglia R, Antonelli M (2010). Diagnostic accuracy of passive leg raising for prediction of fluid responsiveness in adults: systematic review and meta-analysis of clinical studies. Intensive Care Med.

[53] Bentzer P, Griesdale DE, Boyd J, MacLean K, Sirounis D, Ayas NT (2016). Will this hemodynamically unstable patient respond to a bolus of intravenous fluids?. JAMA.

[54] Zhang Z, Xu X, Ye S, Xu L (2014). Ultrasonographic Measurement of the Respiratory Variation in the Inferior Vena Cava Diameter Is Predictive of Fluid Responsiveness in Critically Ill Patients: Systematic Review and Meta-analysis. Ultrasound Med Biol.

[55] Chu H, Wang Y, Sun Y, Wang G (2016). Accuracy of pleth variability index to predict fluid responsiveness in mechanically ventilated patients: a systematic review and meta-analysis. J Clin Monit Comput.

[56] Rivers E, Nguyen B, Havstad S, Ressler J, Muzzin A, Knoblich B, Peterson E, Tomlanovich M (2001). Early Goal-Directed Therapy in the Treatment of Severe Sepsis and Septic Shock. N Engl J Med.

[57] Pearse RM, Harrison DA, MacDonald N, Gillies MA, Blunt M, Ackland G, Grocott MP, Ahern A, Griggs K, Scott R (2014). Effect of a perioperative, cardiac output–guided hemodynamic therapy algorithm on outcomes following major gastrointestinal surgery: A randomized clinical trial and systematic review. Jama.

[58] Monnet X, Marik PE, Teboul J-L (2016). Prediction of fluid responsiveness: an update. Ann Intensive Care.

[59] Godfrey G, Dubrey S, Handy J (2014). A prospective observational study of stroke volume responsiveness to a passive leg raise manoeuvre in healthy non‐starved volunteers as assessed by transthoracic echocardiography. Anaesthesia.

[60] Delerme S, Castro S, Freund Y, Nazeyrollas P, Josse M-O, Madonna-Py B, Rouff E, Riou B, Ray P (2010). Relation between pulse oximetry plethysmographic waveform amplitude induced by passive leg raising and cardiac index in spontaneously breathing subjects. Am J Emerg Med.

[61] Keller G, Cassar E, Desebbe O, Lehot J-J, Cannesson M (2008). Ability of pleth variability index to detect hemodynamic changes induced by passive leg raising in spontaneously breathing volunteers. Crit Care.

[62] Bundgaard‐Nielsen M, Jørgensen CC, Kehlet H, Secher NH (2010). Normovolemia defined according to cardiac stroke volume in healthy supine humans. Clin Physiol Funct Imaging.

[63] Jans Ø, Tollund C, BUNDGAARD‐NIELSEN M, Selmer C, Warberg J, Secher N (2008). Goal‐directed fluid therapy: stroke volume optimisation and cardiac dimensions in supine healthy humans. Acta Anaesthesiol Scand.

